# Rapid Fabrication of Microfluidic Devices for Biological Mimicking: A Survey of Materials and Biocompatibility

**DOI:** 10.3390/mi12030346

**Published:** 2021-03-23

**Authors:** Hui Ling Ma, Ana Carolina Urbaczek, Fayene Zeferino Ribeiro de Souza, Paulo Augusto Gomes Garrido Carneiro Leão, Janice Rodrigues Perussi, Emanuel Carrilho

**Affiliations:** 1 Instituto de Química de São Carlos, Universidade de São Paulo, São Carlos 13566-590, SP, Brazil; huiling@usp.br (H.L.M.); anaurba@yahoo.com.br (A.C.U.); maildafay@gmail.com (F.Z.R.d.S.); paulocleao@gmail.com (P.A.G.G.C.L.); janice@iqsc.usp.br (J.R.P.); 2 Instituto Nacional de Ciência e Tecnologia de Bioanalítica, INCTBio, Campinas 13083-970, SP, Brazil

**Keywords:** organ-on-chip, shear stress, polyester-toner, polyester-vinyl, polystyrene, glass, HUVEC- human umbilical vein endothelial cell line

## Abstract

Microfluidics is an essential technique used in the development of in vitro models for mimicking complex biological systems. The microchip with microfluidic flows offers the precise control of the microenvironment where the cells can grow and structure inside channels to resemble in vivo conditions allowing a proper cellular response investigation. Hence, this study aimed to develop low-cost, simple microchips to simulate the shear stress effect on the human umbilical vein endothelial cells (HUVEC). Differentially from other biological microfluidic devices described in the literature, we used readily available tools like heat-lamination, toner printer, laser cutter and biocompatible double-sided adhesive tapes to bind different layers of materials together, forming a designed composite with a microchannel. In addition, we screened alternative substrates, including polyester-toner, polyester-vinyl, glass, Permanox^®^ and polystyrene to compose the microchips for optimizing cell adhesion, then enabling these microdevices when coupled to a syringe pump, the cells can withstand the fluid shear stress range from 1 to 4 dyne cm^2^. The cell viability was monitored by acridine orange/ethidium bromide (AO/EB) staining to detect live and dead cells. As a result, our fabrication processes were cost-effective and straightforward. The materials investigated in the assembling of the microchips exhibited good cell viability and biocompatibility, providing a dynamic microenvironment for cell proliferation. Therefore, we suggest that these microchips could be available everywhere, allowing in vitro assays for daily laboratory experiments and further developing the organ-on-a-chip concept.

## 1. Introduction

The development of a new in vitro model for investigating cell stimulation and drug screening has become increasingly emergent because the most available assays usually require extended time and/or are costly experiments [1]. The combination of miniaturization and microfluidics has recently exhibited exceptional performance in creating in vitro models that enable mimicking human organ function and provide a better understanding of cell response to extrinsic physical and biochemical stimuli [2]. The microchip with a proper architecture design can emulate a suitable in vitro model and offers unparalleled advantages, including precise control of dynamic stresses and fluid gradients, facile monitoring of cell status and forming a highly structured tissue [3]. Depending on the fabrication process, it is attainable to create a microdevice with design and geometry as necessary using versatile materials, including polydimethylsiloxane (PDMS), glass, paper and polyester films [4,5,6,7]. Additionally, miniaturization reduces operating costs through low consumption of reagents (from nano to microliters of reagents) followed by the possibility of high throughput by parallelization of many devices running tests and assays simultaneously [8]. For this reason, the microchip has been explored as an alternative in preclinical drug trials because of outstanding economic and technological advantages.

The traditional cytotoxicity assays performed under static conditions (e.g., 96-well plates) have explicitly failed to simulate in vivo conditions such as shear stress effects inside microvessel [9]. Microchips with microscale channels can provide a dynamic microenvironment for cell proliferation and allow further investigation of diverse cellular responses and their flow-dependent behaviors, including morphologic changes, cell migration, cytotoxic and biochemical alterations [10]. Some studies reported that either drug compounds or nanoparticles exhibited high shear stress dependency on cytotoxicity when tested under dynamic conditions [11,12,13]. This phenomenon demonstrates the importance of developing a microchip that can impose shear stress on endothelial cells and significantly affecting cellular signaling pathways. In addition, such an environment allows more predictable drug testing stimulation [14].

Soft lithography has mainly been used as a primary microfabrication technique to generate PDMS microchips with microchannels to simulate the shear stress effect [15,16]. This technique employs liquid polymer to cast into a patterned mold. After cross-linking the polymer, the substrate presents micro to nanoscale features [17]. However, replicating and transferring steps require accurate alignment of the parts to produce complex molds with good quality during the fabrication process [18]. Moreover, every new design of a PDMS device demands manufacturing new physical molds, making these microdevices costly and laborious [16]. It is rather challenging to use soft lithography to fabricate microchips when one needs dynamic changes.

The alternative microfabrication approaches featuring excellent flexibility, scalability and versatility are still of great interest to the field of microfluidics. Previous reports have developed microfluidic devices based on the conventional laboratory tools, including print, cut and laminate (PCL) and laser-cutting thin double-sided tape technique that immensely simplified the fabrication process. These on-the-bench tools produced microchannels with sufficient resolution and prompt use in analytical chemistry and biological studies [19,20,21]. In this respect, we aimed to develop microchannel-microchips with efficiency in the simulation of shear stress effect on human endothelial cells by monitoring cell status, viability and attachment area. The interesting feature in our microchips is the use of composite materials by combining biocompatible and transparent substrates, such as polyester-toner, polyester-vinyl, glass, Permanox^®^, polystyrene and double-side adhesive, following by these simple tools or steps like heating-lamination, toner printer and CO_2_ laser cutter. This survey of straightforward procedures affords rapid replication in daily experiments to yield the microchips that are well-sealed against bursting during culture medium perfusion and could potentially aid to widespread cellular biology research at low cost.

## 2. Material and Methods

### 2.1. Laminated Microchip Fabrication

The fabrication of the laminated microchips requires heating, pressure and gluing processes to join multiple layers, creating a composite material, making the microchips stiffer and more stable for assays. Hence, we have experimented with two different microchip fabrication approaches using lamination: polyester-toner (PET) microchip and polyester-vinyl microchip.

**Figure 1:**
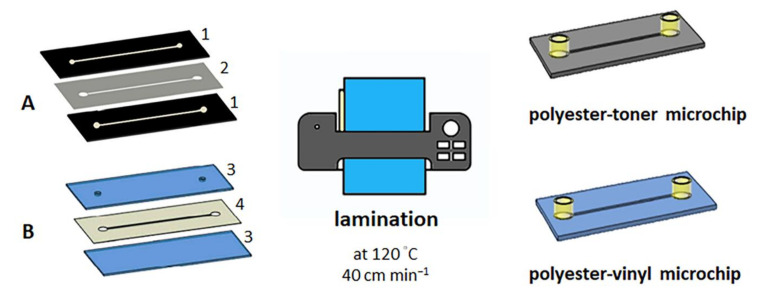
The laminated microchip fabrication is based on using a laminator at 120 °C and 40 cm min^−1^ to join the microchip layers. (**A**) PET microchip (the microchannel dimension is 2 × 2 with the cut channel 5 mm × 0.103 mm) consisting of polyester film with channel toner printed in one side (1) and bare polyester film (2). (**B**) polyester-vinyl microchip with bare polyester film (3) and the middle layer is made of three layers of vinyl adhesives with a cut-through channel (4). The top of the microchips contains a female luer glued to facilitate the silicone hose fitting for microfluidic operation.

The polyester-toner microchip was fabricated according to the PCL approach described by Thompson et al. [19]. Afterward, following a previous publication from our group, we assembled the microchips using the PCL method and cultured endothelial cells under perfusion [20]. The disposable microchips (25 mm × 30 mm) consisted of a microchannel with dimensions of 2 mm × 25 mm × 0.103 mm, width, length and height, respectively. The microchip inlet and outlet (d = 3 mm) in the top layer consisted of punched holes to provide access to the microchannel (Figure 1A). 

The polyester-vinyl microchip has utilized the same previous layout of the microchannel (2 mm × 25 mm). This microchip employed two polyester films (25 mm × 30 mm) treated with oxygen plasma (50 W and 70 mtorr for 2 min) as the top and bottom layers and three vinyl adhesives (3M Scotchcal^TM^ D3000, 25 mm × 30 mm × 0.09 mm) with a cut-off microchannel in CO_2_ laser cutter as the middle layer. These three layers were aligned and laminated together using a laminator (AC 00-1230, Gazela, Brazil) at 120 °C and 40 cm min^−1^. Epoxy resin fixed the female luers on the channel access holes (Figure 1B). The microchannel of all laminated microchips was infused with 50 µL of 10 µg mL^−1^ fibronectin (from bovine plasma, Sigma-Aldrich) in PBS incubated overnight at 4 °C to improve the cell adhesion.

### 2.2. Biocompatible Adhesive Microchip Fabrication

The Biocompatible adhesive microchips consist mainly of three layers: top, middle and bottom, each with a specific function. The top layer allows visualization of cell growth inside the microchannel in optical microscopy. Thus, the selected materials were transparent such as polyester film (20 mm × 50 mm, Data Jet, Brazil), glass slide (25.4 mm × 76.2 mm with a thickness of 1 to 1.2 mm, Perfecta, Brazil), or coverslip (24 mm × 50 mm with a thickness of 0.13 to 0.17 mm, Perfecta, Brazil). 

The bottom layer serves as a bed for the growth of the HUVEC; therefore, the materials with good cell adhesion and growth, such as Permanox^®^ slide (75 mm × 25 mm, Thermo Scientific Nunc, US), polystyrene slide (75 mm × 25 mm, obtained from cutting of 75 cm^2^ cell culture flask, Corning, USA) or glass slide (76.2 mm × 25.4 mm with a thickness of 1 to 1.2 mm, Perfecta, Brazil) treated with 50 µL of fibronectin solution (fibronectin from bovine plasma, Sigma-Aldrich) at 10 µg mL^−1^ prevailed as suitable in this task.

**Figure 2:**
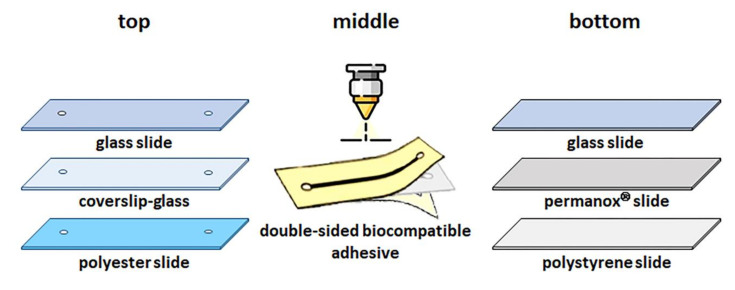
The biocompatible adhesive microchip fabrication is based on using a double-sided biocompatible adhesive to join the top and bottom layers. Top layer: glass slide, coverslip, or polyester). Middle layer: cut double-sided biocompatible adhesive. Bottom layer: glass slide, Permanox^®^, or polystyrene film. The top of the microchip is a female luer glued to facilitate the silicone hose fitting for microfluidic operation.

The middle layer served to seal the top and bottom layers and delimit the microchannel. In this way, a microchannel designed in CorelDRAW^®^15 software (Corel Corp., Canada) with dimensions 2 mm × 25 mm and two circles with 3 mm in diameter to be inlet and outlet was the central microstructure. This layout was cut in the double-sided biocompatible adhesive (ARcare^®^ - 90106, Adhesives Research, Ireland) using a CO_2_ laser cutter (Combat Laser 600) [21]. After cutting, the peeling of the release liners sealed the top and bottom layers. Finally, female luers were glued with epoxy resin on the top layer to access the microchannel inlet and outlet (Figure 2).

The microchannel final dimension of biocompatible adhesive microchips has length and width, 25 mm × 2 mm, respectively. The height was 0.14 mm, corresponding to the thickness of double-sided biocompatible adhesive without the release liners.

### 2.3. Cell Line and Culture Medium

Human umbilical vein endothelial cells (HUVEC) (CRL-2873™; ATCC^®^, Baltimore, MD, USA) were grown in polystyrene cell culture flasks (Corning, New York, NY, USA) with Roswell Park Memorial Institute (RPMI) 1640 medium containing 25 mM HEPES (Sigma-Aldrich, St. Louis, MS, USA) supplemented with 10% fetal bovine serum (FBS, Cultilab, Campinas, Brazil) and 1% mix of antibiotics/antifungals (Sigma-Aldrich, St. Louis, MS, USA). Cells grew at 37 °C in an incubator with 5% CO_2_ and removed from the flask with a trypsin-EDTA solution (Sigma-Aldrich, St. Louis, MS, USA).

### 2.4. Cell Seeding and Cultivation in Microchip

**Figure 3:**
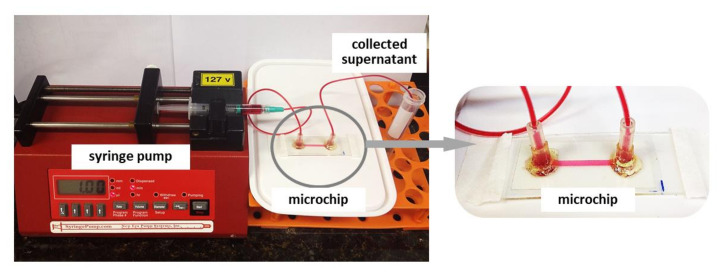
Schematics of the experimental setup of the dynamic microchip system. After cell adhesion to the microchannel for 4 h under static conditions in the incubator (a crucial point in the cells’ ability to resist the subsequent shear stress), the microchip was connected to a syringe pump. The microchip was perfused at a continuous unidirectional flow rate of 2 µL min^−1^ with RPMI-1640 culture medium. A Teflon tubing connected the syringe pump and the microchip through a female luer adapter. The supernatant exited on the opposite access and collected into a sterile Falcon^®^ tube sealed with plastic film (Parafilm^®^ “M”–Laboratory Film). The system was kept in the incubator at 37 °C at 5% CO_2_ during the experiment.

All microchips were sterilized by perfusion with 70% ethanol (Labsynth, Diadema, Brazil) at 5 µL mL^−1^ using a syringe pump (Harvard Apparatus, Cambridge, MA, USA) and exposing it to UV light for 40 min. After that, the microchannel was rinsed three times with sterile PBS to remove ethanol and then three times with culture medium. The HUVEC suspensions at 5 × 10^6^ cells mL^−1^ were injected into the sterilized microchannel. Then, with the inlet and outlet capped, the microchip rested in an incubator (37 °C, 5% CO_2_) for 4 h in order to allow good cell adhesion with the microchip remaining under static conditions. After this period, a semi-confluent and out-stretched cell monolayer formed. Subsequently, a syringe pump perfused the microchannel at a continuous unidirectional flow rate of 2 µL min^−1^ with RPMI-1640 culture medium supplemented with 20% FBS and 1% mix of antibiotics and antifungals. The whole system, including the microchip and the syringe pump (Figure 3), remained in an incubator at 37 °C at 5% CO_2_ during the experiment. We monitored the cell proliferation inside the microchannel using an inverted microscope (CKX41, Olympus, Tokyo, Japan) equipped with a CCD camera (Q-Color5™, Olympus, Tokyo, Japan) and used ImageJ software to calculate the cell attachment area inside the microchannels in all microchips.

### 2.5. Cell Viability

The cell viability was determined using 30 μL of the mixture of fluorescent dyes: acridine orange (AO, Sigma-Aldrich) and ethidium bromide (EB, Sigma-Aldrich) at the ratio 1:1 at 100 mg mL^−1^ in PBS. After 1 min, the cells were washed three times with PBS to remove excess dyes. The microchip was taken immediately to the fluorescence microscope (Olympus BX4) at 460/90 nm excitation filter. The images were obtained by Olympus DP72 camera. The numbers of dead and living cells were quantified by counting the number of green dots (live cells), red- orange dots (dead cells).

### 2.6. Statistical Analysis

At least three replicates of each experiment supplied data for statistical analysis. All results bring the mean ± one standard deviation. One-way ANOVA performed statistical analysis with Tukey test to compare two groups. *P* values of <0.01 and 0.05 indicated statistically significant differences.

## 3. Results and Discussion

In the present study, we employed two main microfabrication processes: lamination and biocompatible adhesive to create microchips with one straight channel, which provides precise fluid modeling and accurate control of cell culture medium flow for HUVEC growth under shear stress condition. The cytotoxicity of materials polyester-toner, polyester film and epoxy glue were assayed in our previous publication and showed no cytotoxicity [20]. In addition, we also have tested the cytotoxicity of vinyl adhesive and double-sided biocompatible adhesive tape (ARcare^®^-90106, Adhesives Research, Limerok, Ireland) by 3-(4,5-dimethyl-2-thiazolyl)-2,5-diphenyl-2-H-tetrazolium bromide (MTT) and found that there are no cytotoxic effects for both materials (data not shown). HUVEC were cultured in a microchannel for up to 24 h. During this time, an optical microscope with high-resolution imaging can easily record the cell morphology changes; thus, the microchips offer a suitable microenvironment for cell attachment, proliferation and counting.

**Figure 4:**
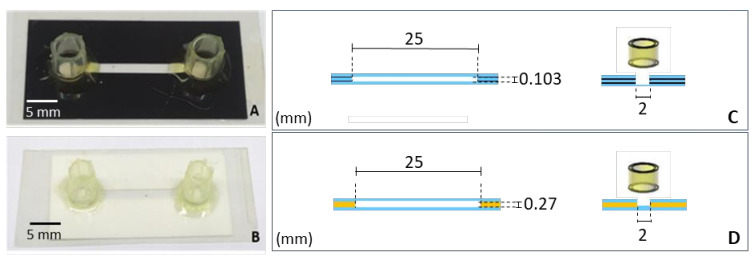
Laminated microchips. (**A**,**C**): Photography of PET microchip and its frontal and transversal section dimensions, respectively. (**B**,**D**): Photograph of vinyl adhesive microchip and its frontal and transversal section dimensions, respectively. The blue layer is polyester films, the black is the toner and the yellow is the vinyl film.

**Figure 5:**
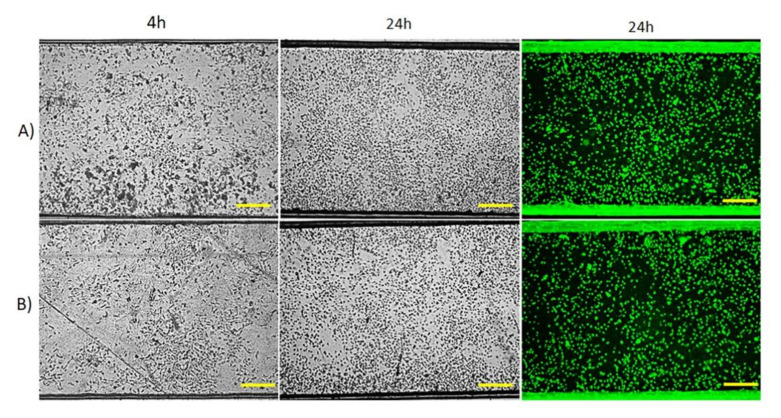
Microchannel of laminated microchips filled with HUVEC at 5 × 10^6^ cells mL^–1^, after 4 h and 24 h of incubation at 37 °C with RPMI-1640 culture medium flow rate 2 µL min^−1^. The cell viability was detected by the mixture of AO/EB staining. Green dots correspond to live cells and red-orange dots correspond to dead cells when present. (**A**) in PET microchip and (**B**) in the polyester-vinyl microchip. Scale bar = 200 µm.

**Figure 6:**
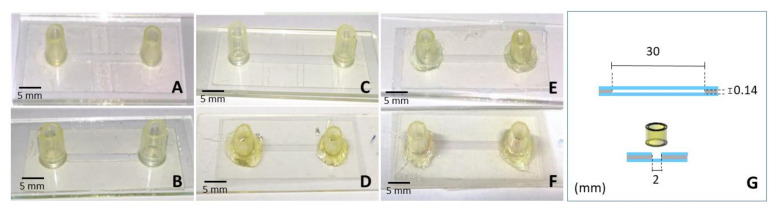
Biocompatible adhesive microchips made of different top-bottom materials: (**A**) glass-glass; (**B**) coverslip-glass; (**C**) glass-Permanox^®^; (**D**) polyester-Permanox^®^; (**E**) glass-polystyrene; (**F**) polyester-polystyrene; (**G**) frontal and lateral section dimensions of biocompatible adhesive microchips illustrating the length (30 mm), the height (0.14 mm) and the width (2 mm) of the channel.

To verify the microchannel dimensions, we introduced distilled water to determine the volume and then calculated the height by width and length. As a result, the dimensions shown in Figure 4, Figure 5 and Figure 6 were confirmed by the obtained volumes of the channels: PET ~5.15 µL, vinyl adhesive ~13.5 µL and all biocompatible adhesive ~8.4 µL. Therefore, the commercial CO_2_ laser system has shown flexibility for allowing fast fabrication of microchip, due to its ability to modify or discard unwanted surfaces by using appropriate laser parameters and feature designs. Moreover, numerous polymer types can be melted and vaporized at high temperatures by focusing a laser beam onto a substrate [22]. Although some factors can affect the cutting quality, such as polymer and machine conditions, including surface finish and over heat-affected corner zone, polyester is one of the polymers with the highest stability during laser cutting; this allows the cutting resolution to be more accurate and suitable to create a channel with a uniform aspect [23,24]. In addition, the physical-chemical properties of the vinyl and polyester films also protect the microchip against liquid leakage and evaporation, meanwhile preventing external contaminations.

### 3.1. Laminated Microchips: Polyester-Toner (PET) and Polyester-Vinyl

The laminated microchip fabrication was based on lamination by heating without material deformation and afforded excellent sealing. The layers like polyester-toner film and vinyl adhesive were employed to build PET and vinyl microchips, respectively. Figure 4 shows the PET microchip and vinyl adhesive microchip and their frontal and transversal section dimensions. Lamination is a versatile tool largely explored in microdevice fabrication on account of its ability to rapidly arrange and produce micro and nanoscale features of various shapes and dimensions for several applications [25]. 

To improve HUVEC adhesion on the microchannel surface, the polyester films utilized in both microchips, PET and polyester-vinyl adhesive, were pretreated with oxygen plasma and fibronectin [26]. Figure 5A shows HUVEC inside PET microchip after 4 h of cell injection and after 24 h of perfusion of RPMI-1640 culture medium using a syringe pump at 2 µL min^−1^, it can be observed that a confluent elongated shape, excellent cell proliferation and filled the microchannel. A similar result was obtained from the polyester-vinyl adhesive microchip (Figure 5B). In addition, HUVEC proliferation on a piece of the polyester film treated with oxygen plasma and fibronectin was performed in the static condition without perfusion and taken as control (Figure S1). We observed that the cell growth had not been affected either on microchips or on a simple substrate placed in a petri dish. The cell viability assessed by AO/EB staining showed that most cells are viable (green dots). There are few dead cells (orange-red dots). The fluorescence microscopy images with incremental magnification of PET microchip and polyester-vinyl adhesive microchip are presented in Figure S2A,B, respectively.

Several studies have demonstrated that lamination of polyester films is suitable for creating new microdevices with intricate design and potential of high-throughput screening, which can be applicable in analytical chemistry, nanotechnology, microfluidics and, recently, organ-on-chip [27,28]. Furthermore, according to the product information, polyester-vinyl can be used to assemble in vitro diagnostics devices and have good results for cell-based assays [29]. These advantages afford this technique to be more practical than other microfabrication techniques. Here, we demonstrated how the combination of polyester film and lamination was easy and fast manufacturing for the rapid prototyping of cell culture assay. The result shows that HUVEC can adhere, proliferate and survive inside of a designed microchip. 

### 3.2. Biocompatible Adhesive Microchips

Microfluidic biochips made with double-sided adhesive tapes (ARcare^®^-90106) using a CO_2_ laser cutter are a very interesting option for fabrication of fast, inexpensive and biocompatible microchips [21]. In this reference, Patko et al. showed that *Salmonella typhimurium* cells could successfully adhere to a transparent optical sensor but lacked data on cell viability and flow effects on them. Since our focus is the development of biomimetic organ-on-a-chip using human cells a survey of low-cost, transparent biomaterials becomes necessary.

The microchannel layout was cut in the double-sided biocompatible adhesive (ARcare^®^-90106) using a CO_2_ laser cutter. The adhesive delimited the area and height of the chamber and joined the top and bottom substrates of the microdevices. Figure 6 shows the biocompatible adhesive microchips and the frontal and transversal section dimensions of biocompatible adhesive microchips.

**Figure 7:**
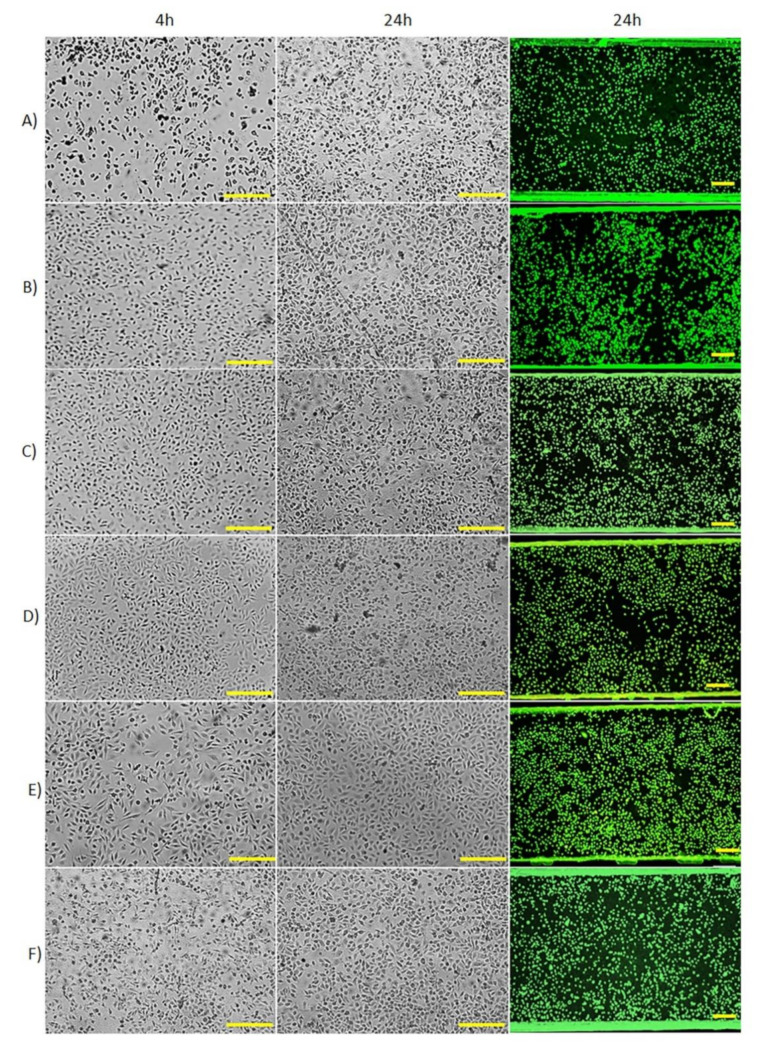
HUVEC (5 × 10^6^ cells mL^–1^) proliferation after 4 h and 24 h of incubation at 37 °C with RPMI-1640 culture medium flow rate at 2 µL min^−1^ in the microchannel of biocompatible double-sided adhesive microchips made with top-bottom layers: (**A**) glass slide-glass slide, (**B**) coverslip-glass slide, (**C**) glass slide-Permanox^®^, (**D**) polyester-Permanox^®^, (**E**) glass-polystyrene slide, (**F**) polyester-polystyrene slide. The cell viability was detected by the mixture of AO and EB staining. Green dots correspond to live cells and red-orange dots correspond to dead cells. Scale bar = 200 µm.

The biocompatible adhesive cut in the laser cutter had a precisely defined microchannel shape in solid materials like plastics and glass, resulting in a robust microchip, which is well sealed without leakage of liquids and resistant to aqueous solution washing. Figure 7 shows HUVEC growth on microchannel of different materials: glass slide-glass slide, coverslip-glass slide, glass slide-Permanox^®^, polyester-Permanox^®^, glass-polystyrene and polyester-polystyrene microchips. The AO/EB staining shows that HUVEC are viable (green dots) in all microchips and the cell viability is above 95%. There are few dead cells (red-orange dots). The fluorescence microscopy images with incremental magnification of the biocompatible adhesive microchips are presented in Figure S2C–H.

When the glass slide composed the bottom layer (Figure 7A,B), the cells remained spherical after 4 h; however, after 24 h of perfusion, the cells turned confluent with an elongated shape. Although the glass showed lower cell adhesion than other surfaces, the glass is one of the materials most widely used for cell culture owing to its optical transparency [30]. Instead of the glass slide at the top, the utilization of coverslip contributed to decrease microchip thickness and facilitate optical microscopy. 

Because of the well-known properties of Permanox^®^, such as excellent cell adhesion, non-toxicity and transparency, we explored it as an alternative for producing low-cost microchips. In Figure 7C,D, HUVEC efficiently adhered to Permanox^®^, even only 1 h after injection (Figure S3). A greater quantity of cells adhered to this chip than to glass slide or polyester microchips treated with oxygen plasma and fibronectin, which adhesion just occurs after 4 h of incubation. Permanox^®^ slide with no extra treatments showed superior results in less time than the other materials; this commercial plastic thrives on promoting cell adhesion in this substrate. As a result, the HUVEC filled 50–60% of the channel after 4 h in the incubator. The microchips remained under perfusion for 24 and 48 h (data not shown) and the cells kept adhered to the channel showing a high survival rate of the cells. We did not experiment with cell cultures over 48 h in the microchannel, because when 5 × 10^6^ cells mL^−1^ was introduced in the microchannel, the cell reached 70% confluence in 24 h and about 90% in 48 h. In this context, longer culture times may cause a sudden drop in cell viability due to the lack of space for cell adhesion. Therefore, we suggest that the long culture times can be applied depending on the initial number of cells and cell type to find the best cell condition for other studies.

We selected polystyrene slides as the substrate for cell adhesion and the glass or the polyester slide as the top part for another combination of biocompatible materials. Figure 7E,F show the HUVEC proliferation in polystyrene substrate. We observed about 20% of cell confluence after 4 h seeding and circa 50% after 24 h. It infers that the polystyrene favored cell growth even under the shear stress condition since this material has already been applied in static in vitro cell culture for many years [31]. 

### 3.3. Cell Attachment Area

**Figure 8:**
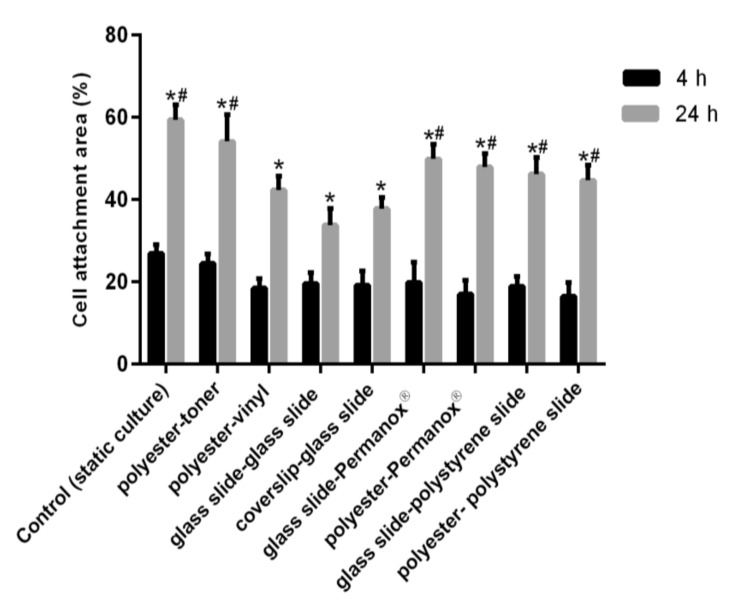
Biocompatibility of all microchips measured by the cell attachment area, which is the percentage occupied by the cells of the total area of the channel. Significant difference * *p* < 0.01 against 4 h. ^#^ *p* > 0.05 no significant difference among the control, polyester-toner, glass slide-Permanox^®^ and polyester-Permanox^®^ microchips in 24 h.

The cell attachment area in the microchannel was quantified using software Image J. Figure 8 shows the comparison of the total area after the cell confluence, a significant cell growth was obtained between 4 and 24 h in all microchips (*p* < 0.01). On the other hand, compared to the control performed under static condition using a plate of 24 wells (Figure S4), there was no significant difference in 24 h among the control, polyester-toner, glass slide-Permanox^®^, polyester-Permanox^®^, glass-polystyrene and polyester-polystyrene microchips (*p* > 0.05). It demonstrates that the bottom layer, polyester treated with oxygen plasma and fibronectin, displayed promising results as the Permanox^®^ and polystyrene for cell attachment. Furthermore, the quantification study also validated that HUVEC grew efficiently in all microchips when these were perfused with culture medium at 2 µL min^–1^ over 24 h. This flow was equivalent fluid shear stress 2.45 dyne cm^–2^ for biocompatible adhesive microchips and 4.48 dyne cm^–2^ for PET microchip and 1.12 dyne cm^–2^ for Polyester-vinyl microchip, according to the calculations [32]. Even under such low shear stress conditions, its effect over 24 h was essential to promote cell proliferation. Therefore, these simple microchips can provide abundant information for drug screening, personalized medicine, metabolic and toxicity studies. In addition, the fluid shear stress is able to remodel cell organization, alter gene and protein expression and influence cell proliferation in the flow perfusion culture compared with the static cell culture [33]. Moreover, we have also performed experiments with the culture of other cell lines in microchannels, such as Vero (kidney epithelial cells extracted from an African green monkey), HEp-2 (human laryngeal carcinoma) and MCF-7 (breast cancer). The proliferation of all those cell lines was as good as HUVEC (data not shown) because the properties of the microchannels surface favoring cell adhesion and growth. 

### 3.4. Comparison of the Cost, Fabrication and Efficiency of Microchips

Table 1 shows some relevant features of the microchips we produced, including costs for microfabrication, the time for manufacturing them and, most importantly, the ability of cell adhesion to the microchip in a short time. A critical feature in the fabrication of these microchips was using the CO_2_ laser cutter that generated precisely cut slides. When comparing the two processes used to bind different materials: lamination or double-sided adhesive, the latter proved an exciting alternative to make microchannel, including advantages of the short time required for fabrication, high reproducibility and low-cost. 

To improve cell proliferation, many compounds like fibronectin, vitronectin, collagen, laminin, fibrin and other growth factors can be used to promote cell adhesion on substrates [34]. However, these reagents are generally expensive and require an additional step for microchip preparation. In our study, the employment of materials that have already been recognized as promising cell adhesion surfaces can significantly reduce the cost and work time. Although polyester is cheaper than other substrates for HUVEC monolayer cell formation, it required a long pre-treatment time with oxygen plasma and fibronectin to be viable. On the other hand, slides like glass, Permanox^®^ and polystyrene have exhibited good cell proliferation and they were reused three times after sterilization with 70% ethanol and UV. Significantly, the commercial Permanox^®^ showed increased high efficiency in cell adhesion, despite the elevated cost.

## 4. Conclusions

In this study, considering the practicality of a CO_2_ laser cutter to cut polymeric materials, we developed two simple and cost-effective techniques: lamination and the stacking of biocompatible double-sided adhesive, to fabricate several microchips with a single straight microchannel. Additionally, for better cell attachment, the polyester surface was treated with oxygen plasma and fibronectin. The results demonstrated that all developed microchips displayed suitable cell attachment and proliferation. These microchips will serve as alternative methods to test compounds under shear stress conditions and lead drug screening to be more reliably predicted in terms of the efficacy and toxicity in humans. We suggest that these platforms be further employed to cultivate other cell lines that feature complex microarchitectures on shear stress effect investigation. 

## Figures and Tables

**Table 1:** Comparative analyses for the cost of materials and the time for fabrication for each type of microchip produced at the laboratory scale capacity.

Microchip *	Cost ($)	Fabrication Time (h)
Polyester-toner ^#^	0.25	4
Polyester-vinyl ^#^	0.30	4
Glass-glass	0.50	1
Coverslip-glass	0.60	1
Glass-Permanox^®^	2.15	1
Polyester-Permanox^®^	2.50	2
Glass-polystyrene	0.75	4
Polyester-polystyrene	1.25	3

* Durability: All chips were reused three times for independent experiments; no further reuse was tested. ^#^ Additional 24 h needed to prepare the channel for cell adhesion.

## Supplementary Materials

The following are available online at https://www.mdpi.com/2072-666X/12/3/346/s1, Figure S1: HUVEC cells proliferation on a piece of polyester film treated with oxygen plasma and fibronectin in the static conditions without culture medium perfusion, which were taken as the control experiment. A: time zero. B: 4 h. C: 24 h. Scale bar = 100 µm; Figure S2: HUVEC (5 × 106 cells mL^–1^) were stained with AO/EB (green dots correspond to live cells and red-orange dots cor-respond to dead cells) after 24 h of incubation at 37 °C with RPMI culture medium flow rate at 2 µL min^−1^ in the microchannel. A) PET microchip and B) polyester-vinyl microchip. The biocompatible double-sided adhesive microchips made with top-bottom layers: C) glass slide-glass slide, D) co-verslip-glass slide, E) glass slide-Permanox^®^, F) polyester-Permanox^®^, G) glass-polystyrene slide, H) polyester-polystyrene slide. The Scale bar = 100 µm; Figure S3: HUVEC cells proliferation under RPMI culture medium flow rate at 2 µL min^−1^ in the microchannel of polyester-Permanox^®^ micro-chip; Figure S4: HUVEC cells proliferating on the 24-well plate (polystyrene surface) in the static condition. A: time zero. B: 4 h. C: 24 h. Scale bar = 100 µm.
