# Supplementary material for: Rapid Fabrication of Microfluidic Devices for Biological Mimicking: A Survey of Materials and Biocompatibility
Source: Micromachines (Basel). 2021 Mar 23;12(3):346. doi: 10.3390/mi12030346 (PMC8005101; doi:10.3390/mi12030346)
Supplement: Supplementary file 1 [file micromachines-12-00346-s001.pdf]

micromachines

Article

Rapid Fabrication of Microfluidic Devices for Biological Mimicking: A Survey of Materials and Biocompatibility

Hui Ling Ma [1,2], Ana Carolina Urbaczek [1], Fayene Zeferino Ribeiro de Souza [1], Paulo Augusto Gomes Garrido Carneiro Leão [1], Janice Rodrigues Perussi [1] and Emanuel Carrilho [1,2,*]

[1] Instituto de Química de São Carlos, Universidade de São Paulo, 13566-590 São Carlos, SP, Brazil huiling@usp.br (H.L.M); anaurba@yahoo.com.br (A.C.U.); maildafay@gmail.com (F.Z.R.d.S.); paulocleao@gmail.com (P.A.G.G.C.L.); janice@iqsc.usp.br (J.R.P.)
[2] Instituto Nacional de Ciência e Tecnologia de Bioanalítica, INCTBio, 13083-970 Campinas, SP, Brazil
* Correspondence: emanuel@iqsc.usp.br, +55 16 3373-944

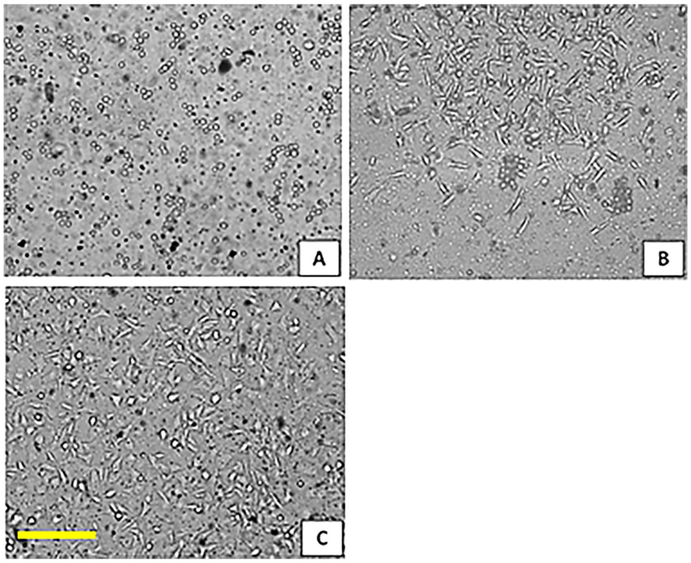

Figure 1. HUVEC cells proliferation on a piece of polyester film treated with oxygen plasma and fibronectin in the static conditions without culture medium perfusion, which were taken as the control experiment. A: time zero. B: 4 h. C: 24 h. Scale bar = 100 µm.

Citation: Ma, H.L.; Urbaczek, A.C.; de Souza, F.Z.R..; Leão, P.A.G.G.C.; Perussi, J.R.; Carrilho, E. Rapid Fabrication of Microfluidic Devices for Biological Mimicking: A Survey of Materials and Biocompatibility. *Micromachines* **2021**, *12*, 346. https://doi.org/10.3390/mi12030346

Academic Editor: András Dér

Received: 22 February 2021
Accepted: 19 March 2021
Published: 23 March 2021

Publisher's Note: MDPI stays neutral with regard to jurisdictional claims in published maps and institutional affiliations.

Copyright: © 2021 by the authors. Licensee MDPI, Basel, Switzerland. This article is an open access article distributed under the terms and conditions of the Creative Commons Attribution (CC BY) license (http://creativecommons.org/licenses/by/4.0/).

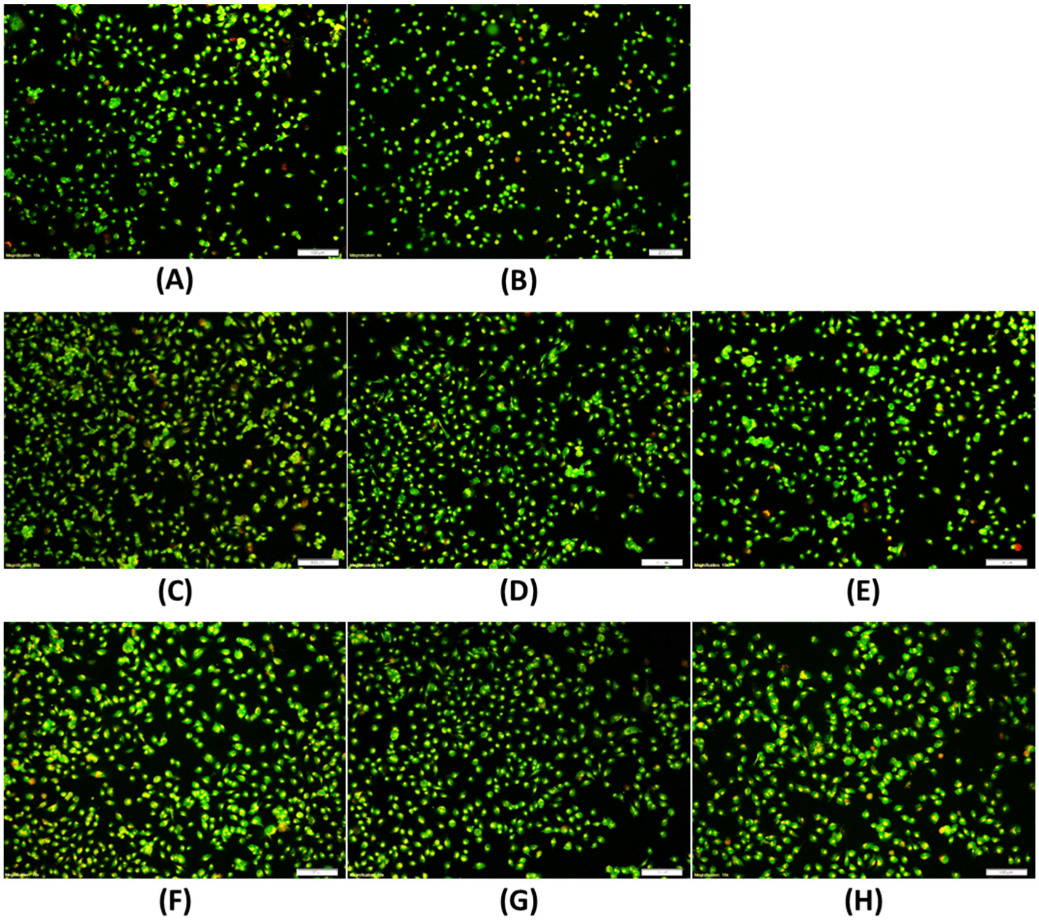

Figure 2. HUVEC (5×10^6 cells mL^{-1}) were stained with AO/EB (green dots correspond to live cells and red-orange dots correspond to dead cells) after 24 h of incubation at 37 °C with RPMI culture medium flow rate at 2 μL min^{-1} in the microchannel. A) PET microchip and B) polyester-vinyl microchip. The biocompatible double-sided adhesive microchips made with top-bottom layers: C) glass slide-glass slide, D) coverslip-glass slide, E) glass slide-Permanox®, F) polyester-Permanox®, G) glass-polystyrene slide, H) polyester-polystyrene slide. The Scale bar = 100 μm.

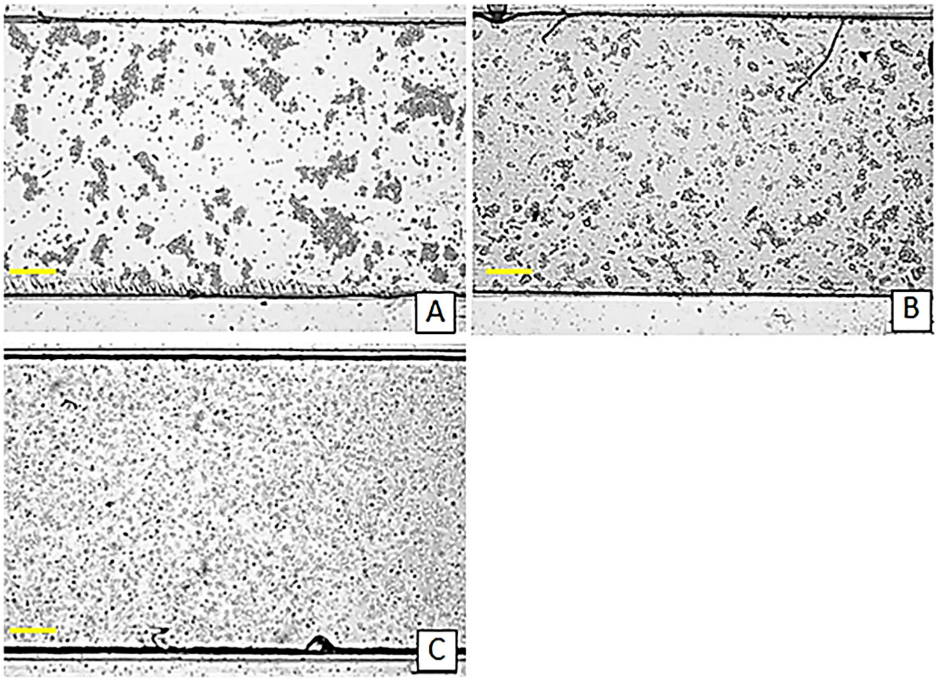

Figure 3. HUVEC cells proliferation under RPMI culture medium flow rate at 2 μL min^{-1} in the microchannel of polyester-Permanox® microchip.

A: 1 h B: 4 h. C: 24 h. Scale bar = 200 μm.

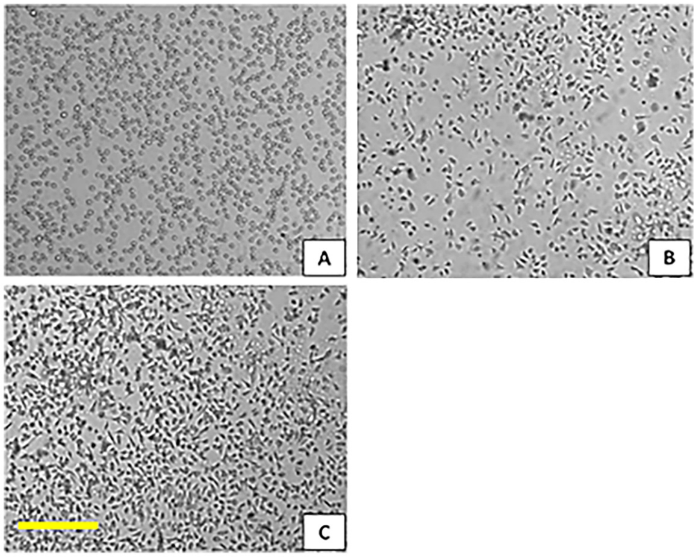

Figure 4. HUVEC cells proliferating on the 24-well plate (polystyrene surface) in the static condition. A: time zero. B: 4 h. C: 24 h. Scale bar = 100 μm.
